# Nutritional Status of Patients With Leprosy Attending a Tertiary Care Institute in North India

**DOI:** 10.7759/cureus.23217

**Published:** 2022-03-16

**Authors:** Rashmi Jindal, Prateek Nagrani, Payal Chauhan, Yashwant S Bisht, Sheenam Sethi, Samarjit Roy

**Affiliations:** 1 Dermatology, Himalayan Institute of Medical Sciences (HIMS), Dehradun, IND; 2 Dermatology, All India Institute of Medical Sciences (AIIMS) Bilaspur, Bilaspur, IND

**Keywords:** bmi, assessment, predisposition, nutrition, leprosy

## Abstract

Introduction

Leprosy is a chronic, infectious disease resulting in significant physical and social morbidity. This study aims to assess the nutritional status of patients with leprosy.

Methods

Treatment-naïve leprosy cases seen over two years at a tertiary care center were recruited in this prospective observational study. Demographic details, type of leprosy, and presence of reactions and disabilities were recorded. Nutritional assessment was done by calculating the body mass index (BMI) and measuring the hemoglobin, serum iron, serum albumin, and serum cholesterol.

Results

Fifty patients were recruited with a mean age of 40.5 (15.3) years. Most patients (60%) had a normal BMI of 18.5-24.9, and 14% were underweight. Two-thirds of the patients had low hemoglobin, with 18 (36%) having low serum iron. A statistically significant association was observed between low serum iron and the multibacillary form of leprosy.

Conclusion

A nutritional assessment should be included in the initial evaluation of leprosy.

## Introduction

Leprosy, like tuberculosis, is perceived as a disease of poverty. This is reflected by the fact that the significant burden of disease is in underdeveloped countries. Indian figures for the year 2019-20 state an annual case detection rate of 8.13 per 100000 population with 1,14,451 new cases detected [1]. At the national level, substantial work is done to detect cases early and treat them in time to decrease disease transmission. Factors predisposing to the development of disease must be identified. The most evident predisposing factors are poverty, under-nutrition, food shortage, food insecurities, and lack of food diversities. Leprosy, with its associated physical and social morbidity, results in economic dislocation leading to malnutrition. This might initiate a self-perpetuating vicious cycle with leprosy resulting in malnutrition and vice-versa. Despite this being common knowledge, the supporting literature is scarce. This study aims to elucidate the nutritional status of leprosy patients and assess its effect on the type of leprosy, reaction states, and deformities. This knowledge will be helpful in future policy making to reduce the disease burden and associated morbidity.

## Materials and methods

Study design

This was a prospective, observational study.

Inclusion criteria

Newly diagnosed treatment naïve cases of leprosy attending the dermatology outpatient department of a tertiary care institute in Uttarakhand, from January 1, 2019, to December 31, 2020, were included after obtaining written informed consent and institutional ethics committee approval (HIMS/RC/2019/54). The diagnosis of leprosy was confirmed by clinical, bacilloscopic, and histopathological examination.

Exclusion criteria

Patients under treatment or defaulters and those with co-morbidities (diabetes, alcoholism) were excluded. Patients on systemic steroids were also excluded.

Study protocol

A structured case reporting form was used to record the demographic details and clinical details, including the type of leprosy (Using the WHO and Ridley Jopling classification systems), presence of deformities, and reactions [2-3]. Nutritional assessment was done by calculating the body mass index (BMI) and measuring the hemoglobin, serum iron, serum albumin, serum cholesterol, and C-reactive protein (CRP) levels. WHO criteria were used for nutritional classification of subjects based on BMI, with a value of <18.5, 18.5-24.9, and >25.0 labeled as underweight, normal weight, and overweight, respectively. The reference laboratory range of biochemical parameters was used to identify patients with deficiencies [4]. Serum cholesterol of less than 160 mg/dl was considered low [5].

Statistical analysis

Statistical analysis was done using the Statistical Package for the Social Sciences (SPSS) version 22 (IBM Corp., Armonk, NY). Those with low BMI, serum iron, serum albumin, and serum cholesterol were compared with those with normal values using the chi-square test. Comparison of means between tuberculoid and lepromatous leprosy patients was made using an independent sample t-test. P < 0.05 was considered statistically significant. Appropriate supplements were prescribed to patients having specific deficiencies, and they were further referred to a physician for an appropriate workup to delineate the underlying cause.

## Results

A total of 104 patients with leprosy were started on multidrug therapy during the two-year study period (58 in 2019 and 46 in 2020). Fifty patients were recruited in the study as the rest (54) had received some form of anti-leprosy treatment in the recent past. The mean age of the patients was 40.5 (15.3) years and the male to female ratio was 3.1 (Table 1).

**Table 1:** Demographic details, clinical profile, and nutritional assessment of patients with leprosy (n=50) TT: tuberculoid; BT: borderline TT; BB: borderline; BL: borderline lepromatous; LL: lepromatous; PB: paucibacillary; MB: multibacillary

Mean age	40.5 (15.3) years
Median age	40 years
	No. (%)
Age range (years)	
≤15	0
16-30	17 (34)
31-45	13 (26)
>45	20 (40)
Gender	
Male	38 (76)
Female	12 (24)
Type of leprosy (RJ^*^ classification)	
TT	0
BT	12 (24)
BB	0
BL	21 (42)
LL	17 (34)
WHO categories	
PB	7 (14)
MB	43 (86)
Reaction state	
Noreaction	22 (440
Type 1	13 (26)
Type 2	15 (30)
Deformities: None, Grade 1, Grade 2	7 (14) 30 (60) 13 (26)
BMI	
Under weight (<18.5)	7 (14)
Normal weight (18.5-24.9)	30 (60)
Over weight (>25.0)	13 (26)
Hemoglobin (g/dl)	
Males <14 g/dl (n=38)	25
Females <12 g/dl (n=12)	8
Total (n=50)	33 (66)
Hemoglobin <10g/dl	8 (16)
Serum iron <41 μg/dl	18 (36)
Serum albumin <3.5 g/dl	14 (28)
Serum cholesterol <160 mg/dl	31 (62)
C-reactive protein >6mg/l	14 (28)

Borderline lepromatous leprosy was diagnosed in 21 (42%) patients, and 17 (34%) had lepromatous leprosy. Forty-three (86%) patients had multibacillary leprosy. Reaction states were identified in 28 (56%) patients, 13 (26%) had a type 1 reaction and 15 (30%) had a type 2 reaction. Deformities were seen in 43 (86%) patients, with 13 (26%) having grade 2 deformities. Most patients had a normal BMI (30, 60%); however, in seven (14%), it was lower than 18.5. Two-thirds of the patients had low hemoglobin, with 18 (36%) having low serum iron. However, hemoglobin levels less than 10 g/dl were observed in eight (16%) patients. Serum albumin and cholesterol were low in 14 (28%) and 31 (62%) subjects, respectively. CRP was raised in 14 (28%) patients. Statistical analysis was performed to assess the effect of nutritional status on the type of leprosy, development of reactions, and presence of deformities using the chi-square test (Table 2).

**Table 2:** Effect of nutritional status on type of leprosy and development of reactions and deformities in patients with leprosy PB: paucibacillary; MB: multibacillary

Nutritional parameter	BMI <18.5	Hemoglobin <14 g/dl males and <12 g/dl females	Serum Iron <41 μg/dl	Serum albumin <3.5 g/dl	Serum Cholesterol <160 mg/dl
Reactions					
Absent	3	13	9	6	11
Present	4	20	9	8	20
p-value	0.95	0.36	0.52	0.91	0.12
Deformities					
Absent	2	4	2	2	5
Present	5	29	16	12	26
p-value	0.23	0.59	0.66	0.97	0.57
WHO category					
PB	2	4	0	2	3
MB	5	29	18	12	20
p-value	0.23	0.59	0.03	0.97	0.26

A statistically significant association was seen between low serum iron (<41 μg/dl) and multibacillary leprosy (p=0.03). Comparison of means between tuberculoid and lepromatous leprosy revealed a statistically higher C-reactive protein value in lepromatous leprosy (p=0.014, Table 3).

**Table 3:** Comparison of mean BMI, hemoglobin, serum iron, serum albumin, serum cholesterol, and C-reactive protein between tuberculoid and lepromatous leprosy

Mean	Tuberculoid (n=12)	Lepromatous (n=38)	p-value
BMI	22.8	23.0	0.92
Hemoglobin (g/dl)	12.5	12.4	0.98
Serum iron (μg/dl)	75.9	58.9	0.27
Serum albumin (g/dl)	3.9	3.8	0.86
Serum cholesterol (mg/dl)	152.1	160.1	0.56
C-reactive protein mg/l	1.4	7.3	0.014

## Discussion

The WHO Global Leprosy strategy 2021-2030 "towards zero leprosy" aims for a leprosy-free world [6]. In addition to early diagnosis and adequate treatment, emphasis is also on reducing disease transmission. Nutritional status contributes significantly to regulating immune response against various pathogens, and Mycobacterium leprae should be no exception. Undernutrition has been purported as an essential risk factor predisposing one to develop leprosy. Further, it might affect response to treatment. Nutritional assessment of leprosy patients and their families should form part of the initial evaluation to identify specific deficiencies for index cases and at-risk contacts. The main difficulty in analyzing the available literature in this context is the use of different tools as biomarkers of nutritional status by different researchers. The most widely used is body mass index (BMI). Among the serum markers, serum albumin, iron, transferrin, zinc, vitamin A, and vitamin E have been used. Rao et al. found decreased levels of vitamin A, vitamin E, and zinc in patients with lepromatous leprosy compared to healthy controls [7]. They also found an association between undernutrition and the development of deformities. Diffey et al. reported that cured leprosy index cases with deformity were more undernourished than index cases without deformity [8]. The present study was conducted to assess the nutritional status of treatment-naïve leprosy patients. However, due to financial constraints, only assessment of BMI, hemoglobin, serum albumin, serum cholesterol, serum iron, and C-reactive protein could be performed.

Sixty percent of patients had a normal BMI, and 14% were undernourished. Surprisingly, 26% were overweight or obese. Similar findings of normal BMI in most patients with a fraction having obesity were reported from Brazil [9]. Another study from the same region reported obesity in leprosy patients to be 60% [10]. In both of these studies, there appeared no association between reactional states and BMI. BMI failed to correlate with the presence of reaction and deformities or multibacillary disease in the reported study (p>0.05). A similar study recruiting female patients from Indonesia also could not establish an association between bad nutrition and leprosy (p=0.008, OR=0.422) [11]. A previous Indian study reported under-nutrition (BMI<18.5) to be more common in leprosy patients than controls with a significant association between WHO grade 2 disability and under-nutrition; however, no association was established between the number of skin lesions and bacillary load [12].

A reversal of albumin/globulin ratio had been reported in studies done during the late 1990s, with most revealing a gradual decline of serum albumin over the immunological spectrum from tuberculoid to lepromatous disease [13-14]. Low serum albumin has been correlated with the poor condition of the patients and a high bacillary index [15]. Further, there are also reports of a fall in serum albumin levels when patients develop type-2 reactions; this has been hypothesized to be because of interleukin-6-mediated hemodilution [14]. Serum albumin levels lower than 3.5 g/dl were seen in 28% of patients in our study. However, the mean levels remained similar across the tuberculoid and lepromatous spectrum.

Further, no association could be established between low serum albumin values and the presence of reactions and deformities or multibacillary disease (p>0.05). Most patients with leprosy with plantar ulcers were found to have normal serum albumin levels by Oliveria et al. [16]. Thus, studies from diverse regions have varied results, with some reporting association between leprosy and low serum albumin and others failing to do so. Further, a high C-reactive protein can also lower serum albumin. In the reported study, half of the patients with high CRP had low serum albumin.

Evaluation of hematological indices in patients with leprosy suggests a high propensity for anemia and low serum iron levels. The fall in hemoglobin and serum iron appears to be marked in lepromatous leprosy as compared to tuberculoid leprosy [17-19]. In the reported study similar findings were seen too, with 33 (66%) patients having hemoglobin considered low for their age and gender. However, hemoglobin less than 10g/dl was noted in eight (16%) patients. A significant proportion (18, 36%) of patients also had low serum iron levels. Low serum iron levels correlated significantly with multibacillary disease (p=0.03). Thus either multibacillary patients had decreased erythropoiesis secondary to the infiltration of bone marrow by acid-fast bacilli or leprosy patients with anemia were unable to limit bacterial multiplication. Anemic lepromatous leprosy patients have shown to have a blunted erythropoietin response compared with controls having non-inflammatory anemia [20].

Serum cholesterol values less than 160 mg/dl have been shown to reflect malnutrition. A significant proportion (62%) of our patients had low serum cholesterol. There appears to be a diversion of lipoproteins from hepatocytes towards macrophages, resulting in low serum lipoprotein levels [21]. Lepromatous leprosy patients have low levels of high-density lipoprotein-cholesterol, and patients with erythema nodosum leprosum have low total serum cholesterol [21]. However, contradicting studies reporting normal serum cholesterol throughout the leprosy spectrum also exist [22].

Limitations

A significant limitation of the present study is the absence of a control population. Further, other causes of nutritional deficiencies like iron deficiency anemia could not be ruled out. Small sample size and arbitrary selection of biochemical markers based on the institutional availability and financial constraints are the other limitations.

## Conclusions

Thus, leprosy and undernutrition could be correlated in more than one way, and whether undernutrition is the cause or effect of leprosy remains to be elucidated. There is a need to develop standardized case-control as well as cohort studies to answer this question. However, it appears prudent to assess the nutritional status of new/under-treatment/released from treatment patients to take corrective measures. Minimum biochemical markers necessary to measure nutrition must be delineated to decrease the cost of therapy in resource-poor settings. We recommend the inclusion of BMI measurement and the assessment of hemoglobin, serum iron, serum albumin, and serum cholesterol in the initial assessment of a leprosy patient, with sequential follow-up after the initiation of treatment.
